# The impact of developmental stage, tissue type, and sex on DNA double-strand break repair in *Drosophila melanogaster*

**DOI:** 10.1371/journal.pgen.1011250

**Published:** 2024-04-29

**Authors:** Elizabeth L. Graham, Joel Fernandez, Shagun Gandhi, Iqra Choudhry, Natalia Kellam, Jeannine R. LaRocque

**Affiliations:** Department of Human Science, School of Health, Georgetown University Medical Center, Washington, District of Columbia, United States of America; Columbia University, UNITED STATES

## Abstract

Accurate repair of DNA double-strand breaks (DSBs) is essential for the maintenance of genome integrity, as failure to repair DSBs can result in cell death. The cell has evolved two main mechanisms for DSB repair: non-homologous end-joining (NHEJ) and homology-directed repair (HDR), which includes single-strand annealing (SSA) and homologous recombination (HR). While certain factors like age and state of the chromatin are known to influence DSB repair pathway choice, the roles of developmental stage, tissue type, and sex have yet to be elucidated in multicellular organisms. To examine the influence of these factors, DSB repair in various embryonic developmental stages, larva, and adult tissues in *Drosophila melanogaster* was analyzed through molecular analysis of the DR-*white* assay using Tracking across Indels by DEcomposition (TIDE). The proportion of HR repair was highest in tissues that maintain the canonical (G1/S/G2/M) cell cycle and suppressed in both terminally differentiated and polyploid tissues. To determine the impact of sex on repair pathway choice, repair in different tissues in both males and females was analyzed. When molecularly examining tissues containing mostly somatic cells, males and females demonstrated similar proportions of HR and NHEJ. However, when DSB repair was analyzed in male and female premeiotic germline cells utilizing phenotypic analysis of the DR-*white* assay, there was a significant decrease in HR in females compared to males. This study describes the impact of development, tissue-specific cycling profile, and, in some cases, sex on DSB repair outcomes, underscoring the complexity of repair in multicellular organisms.

## Author summary

DNA double-strand breaks (DSBs) are toxic lesions that threaten genome integrity. If not repaired accurately and efficiently, this damage may lead to cell death or tumorigenesis. Cells are able to repair DSBs by two major pathways: non-homologous end joining (NHEJ) and homologous recombination (HR). While factors such as cell cycle phase and age impact the repair pathway choice between NHEJ and HR, understanding the implications of repair in multicellular organisms, which contain heterogeneous tissues at different developmental stages is less clear. Furthermore, the impact of sex on repair in different tissues also remains to be elucidated. We have investigated these factors using an established DSB repair assay to measure repair outcomes in various tissues, developmental stages, and both sexes in *Drosophila melanogaster*. We found that repair by HR is favored in tissues and developmental stages that contain cells exhibiting the canonical cell cycle. While this was consistent in both males and females in most tissues, females suppress HR repair in their premeiotic germline. Our results demonstrate the complexity of repair pathway choice in multicellular organisms and underscores the impact of sex on DSB repair.

## Introduction

To maintain genome integrity, the repair of DNA double-strand breaks (DSBs) is critical, as genomic instability is a hallmark of cancer [1]. DSBs are particularly deleterious lesions because they involve a break on both strands of the double helix. Not only is transcription repressed when a DSB occurs near a transcription site [2], but unrepaired DSBs can also result in apoptosis, mutations, tumorigenesis, premature aging, and genetic rearrangements [3].

There are two major pathways that repair DSBs: non-homologous end-joining (NHEJ) and homology-directed repair (HDR), which includes both single-strand annealing (SSA) and homologous recombination (HR). In NHEJ, the broken DNA ends are directly ligated. This can lead to the restoration of the original sequence or may involve processing of the DSB ends, resulting in insertions or deletions (indels) at the break site [4]. Repair via NHEJ can therefore be error prone.

HDR requires extensive 5’ to 3’ end resection at the break site. 5’ to 3’ end resection occasionally reveals repetitive sequences on either side of the DSB. When these repeats are revealed, they can anneal to each other and facilitate SSA repair [5]. Since the nucleotides between the repetitive sequences are lost following SSA, this repair pathway is also error prone. In contrast, repair of DSBs by HR is considered error free because the lesion is restored using an unbroken homologous donor sequence as a template for repair [6]. There are two main pathways for HR repair: synthesis-dependent strand annealing (SDSA), predominant in mitotically-dividing cells [7], and double-strand break repair (DSBR), common in meiotically dividing cells, as DSBR generates crossover events that are required for proper segregation of homologous chromosomes during meiosis [8].

In both DSBR and SDSA, 5’to 3’ end resection is followed by Rad51-dependent strand invasion by the DNA end on the donor template [6], which can be on either a homologous chromosome or a sister chromatid. Preference has been demonstrated for the sister chromatid as the template for repair [9–11]. Strand invasion is followed by repair synthesis. In SDSA, the newly synthesized strand dissociates, allowing the DNA ends to anneal, ligate, and create a noncrossover product that may include gene conversion [6,12–14]. DSBR involves second end-capture to create a double Holliday junction (dHJ). Depending on how the dHJ is resolved by endonucleases, the product can be either a crossover or a noncrossover [8,14] that may also include gene conversion.

Repair pathway choice is regulated by multiple factors, including age [15], organism [16], and chromatin state [17]. Generally, repair by NHEJ predominates in the G1 phase of the cell cycle, whereas HR is predominant in S and G2 phases when a sister chromatid is present as a template for repair [18,19]. The influence of cell cycle on repair pathway choice has been well-demonstrated in single-cell systems like yeast [20] and human cells [21]. However, multicellular organisms consist of a heterogeneous population of dividing and nondividing cells, which may impact DSB repair outcomes depending on the tissue type. Thus, despite what is known about repair pathway choice, the impact of developmental stage and tissue type on DSB repair in multicellular organisms remains unclear, as cell cycle differs across these variables.

Additionally, current studies have failed to explore the impact of sex on DSB repair. The majority of biomedical and clinical research has occurred using male subjects [22]. Although the biological sciences have included more females in research studies over the past ten years, there has been no significant increase in the use of sex as a variable for data analysis [23]. Importantly, conclusions made from male subjects are not always concordant with those from females [22,24]. This is especially true in eukaryotes such as *Drosophila*, where most of the transcriptome is regulated in a sex-specific manner [25] and where females perform meiotic recombination while males do not [26]. There are also several species that demonstrate sex-specific morphs, or characteristics that are displayed in only one sex [27]. Thus, analyzing the influence of sex on DSB repair pathway choice in different tissue types is critical in determining the factors that influence DSB repair.

*Drosophila melanogaster* provide a unique opportunity to study the impact of developmental stage, tissue type, and sex on DSB repair pathway choice. *Drosophila* embryos and larval tissues maintain different cell cycle profiles depending on their stage in development [28], and adults contain somatic and germline tissues that may also impact DSB repair outcomes. In addition, as a multicellular organism, *Drosophila* offer the ability to directly address the impact of sex on DSB repair outcomes in a variety of tissues. Using an established DSB repair reporter assay, DR-*white* [12], we investigated the impact of developmental stage, tissue type, and sex on DSB repair pathway choice by analyzing DSB repair in embryos and various cycling and non-cycling larval and adult tissues of both sexes. Our data indicate the importance of developmental stage and tissue type—and thus, cell cycle—in repair pathway choice. The data also indicate sex as an important variable for repair and call for the examination of sex as a factor in determining repair pathway choice in other tissue types as well.

## Materials and methods

### Drosophila stocks

All flies were kept on standard NutriFly Bloomington Formulation medium (Genesee Scientific, San Diego, CA) and maintained at 25°C with 12-hour light/dark cycles. *Drosophila* were manipulated with standard genetics. The DR-*white* stock was previously described [12]. The *I-SceI* transgene source was either *heat-shock protein 70* (*hsp70*) inducible I-*Sce*I transgene (*hsp70*.*I-SceI*) [29] or an *I-SceI* transgene with *Drosophila ubiquitin* promoter (*Ubi*::*I-SceI*) [30].

### DSB induction and sample collection

The DR-*white* assay was previously described [12]. Briefly, the assay contains two nonfunctional direct repeats of the *white* gene (*Sce*.*white* and *iwhite*), as well as a *yellow* transgene (*y*+) for phenotypic tracking and an attB site for stable integration on chromosome *2*. *Sce*.*white* contains an I-SceI endonuclease recognition sequence and contains a premature stop codon. Downstream of *Sce*.*white*, the *iwhite* sequence serves as a donor sequence for homologous recombination (HR) repair. HR repair results in gene conversion of the I-SceI recognition sequence to a wild-type *Sac*I sequence, resulting in the wild-type *white* sequence. Repair by single-strand annealing (SSA) results in annealing of the repetitive *white* sequences and loss of the intervening *y+* transgene. The SacI site is present in these products due to the repeated SacI-recognition nucleotides that persist at the insertion site of the original I-SceI recognition sequence, as described [12].

For experiments with DSB induction and repair in larvae and adults, females containing DR-*white* were crossed to males containing *heat-shock protein 70* (*hsp70*) inducible *I-SceI* transgene (*hsp70*.*I-SceI*). For premeiotic germline and whole fly analyses, 0–3 day old progeny of this cross were heat-shocked at 38°C for 1 hour to induce *I-SceI* expression. Heat-shocked progeny were aged to adults and either flash-frozen using dry ice and 100% ethanol for downstream molecular analysis of whole flies, or crossed out as described below for phenotypic analysis of germline repair events. Non-heat-shocked samples for whole flies were similarly collected and processed for downstream molecular analysis of whole flies; these samples served as a control to analyze leaky expression of the *hsp70*.*I-SceI* transgene.

For adult brain tissue collection, adults containing DR-*white* and *hsp70*.*I-SceI* were heat-shocked at 37.5°C for 1 hour. Flies were collected 24 hours after heat shock and heads were removed. Samples were flash frozen immediately after removal using dry ice and 100% ethanol. Two to three independent experiments with at least five experimental replicates each were pooled for each data set. Non-heat-shocked samples for adult heads were similarly collected and processed; these samples served as a control to analyze leaky expression of the *hsp70*.*I-SceI* transgene. For larval brain and salivary gland samples, third-instar larvae containing DR-*white* and *hsp70*.*I-SceI* were heat-shocked at 38°C for 1 hour, aged 24 hours, and dissected in minimal Ringer’s solution (182 mM KCl, 46 mM NaCl, 3 mM CaCl_2_, and 10 mM Tris-Cl, pH adjusted to 7.2 with 1N HCl) using an adapted method [31], and flash-frozen using dry ice and 100% ethanol for downstream analysis. Two to four independent experiments with at least five experimental replicates each were pooled for each data set.

For DSB induction in embryos, females containing an *I-SceI* transgene with *Drosophila ubiquitin* promoter (*Ubi*::*I-SceI*) were crossed to males containing DR-*white*. Constitutive expression allowed for break formation upon fertilization utilizing maternal deposition of *I-SceI* transcripts. Embryos from this cross were collected on grape-juice agar plates between 0–3, 3–6, and 6–20 h post-deposition. Pooled embryos (~30-100/sample) were flash frozen using dry ice and 100% ethanol upon collection. Three to four independent experiments with at least three replicates each were pooled for each age group.

### Germline DSB repair analysis

To perform analyses of premeiotic germline events, heat-shocked F1 DR-*white*/*hsp70*.*I-SceI* progeny were aged to adults and crossed to *y w* tester flies of the opposite sex. F2 progeny, representing individual germline events, were phenotypically analyzed [12,32]. DR-*white* flies have white eyes and brown bodies, a phenotype that remains if no double-strand break (DSB) occurs or if repair occurs by either intersister HR or NHEJ with or without indels. Since HR restores the wild-type *Sac*I sequence at the break site, leading to expression of the functional *white* gene (*w*+), HR flies have red eyes. Yellow-bodied flies with white eyes indicate repair by SSA due to deletion of the *y*+ transgene or a mitotic crossover.

For the male germline, each sample (*n*) included one single male crossed to five tester females in a single vial. In the female germline, each sample (*n*) included two females crossed to five tester males in a single vial for cross productivity purposes. F2 progeny were scored for phenotypic analysis as described above for 10 days post eclosure. Samples (*n*) are presented as the number of vials scored. For all germline experiments, each assay was performed as two to three independent experimental replicates with at least *n* = 14 samples in each, and total samples were combined.

### Genomic DNA extraction

Genomic DNA from all samples used in Tracking across Indels by DEcomposition (TIDE) analysis was extracted following the protocol modified as described previously [9]. Samples (*n* = 2 larval brains, 4–5 wing imaginal discs, 1 pair of larval salivary glands, 1 adult head, or 1 whole fly) were homogenized in Buffer A (50 μL; 100 mM Tris-Cl pH 7.5, 100 mM EDTA, 100 mM NaCl, 0.5% SDS) before a 30-minute incubation at 65°C. Buffer B (100 μL; 1.4 M potassium acetate, 4.3 M LiCl) was added and the samples were incubated on ice for 30 minutes. After incubation, the samples were centrifuged at 13,200 rpm for 15 minutes at 4°C. 100 μL of 100% isopropanol was added to the supernatant, and the samples were centrifuged at room temperature (13,200 rpm, 10 minutes). DNA pellets were washed with cold 70% ethanol, and the samples were centrifuged at 4°C for 5 minutes. The samples were allowed to air dry for 20–30 minutes before resuspending the pellet in 20 μL of nuclease-free water.

### Sce.white polymerase chain reactions

Genomic DNA concentrations and purity ratios were determined using a NanoDrop One spectrophotometer. 100 ng of genomic DNA was used to perform PCR reactions using the SapphireAMP Fast PCR Master Mix (Takara). Primers 5’AGCTTTCGCTCAGCAAATGTC (forward) and 5’ GTGACTCTGCGACGTATTTAT (reverse) or 5’ GCGTGGATCAGGTGATCCAG (forward) and 5’ ATCTTAAGCCATCGTCAGTTG (reverse) amplified the *Sce*.*white* sequence in the DR-*white* assay using the Touchdown 30 protocol described previously [9]: 94°C, 2 min; [94°C, 30s; 66°C touchdown (-0.5°C per cycle), 30s; 72°C, 30s]16x; [94°C, 30s; 58°C, 30s; 72°C, 30s]20x; 72°C, 5 min. PCR products were run on a 1% agarose gel. Samples with visible amplicons were purified using Wizard SV PCR Clean-Up System (Promega) and eluted in 25 μL water. 40–100 ng of purified samples were sent for Sanger sequencing (Azenta) using sequencing primer 5’-GAGCCCACCTCCGGACTGGAC.

### TIDE analysis

*Sce*.*white* sequences (.abi files) were analyzed by the TIDE method, as previously described and customized for the DR-*white* assay [33,34]. Using the known *Sce*.*white* sequence around the I-SceI DSB site, HR is identified as a 23 bp deletion that restores the wild-type *SacI* sequence (GAGCTC). Insertions and deletions up to 35 nucleotides are scored as NHEJ with indels events (called “NHEJ” herein). Outputs at zero indels are defined as “no DSB”, since the I-SceI recognition sequence has been maintained and no detectable repair events have occurred. Sequencing chromatograms for each sample were analyzed for their quality, and sequences containing high background were excluded. The proportion of HR and NHEJ determined using TIDE are presented as a percentage of total detectable DSB repair events (i.e., +/- 35 indels representing HR and NHEJ events only; 0 indels representing “no DSB” events are excluded). Absolute HR repair is calculated as the absolute proportion (%) of HR out of all events (i.e., +/- 35 indels representing HR and NHEJ events and including 0 indels representing “no DSB” events).

### Y chromosome genotyping PCR

Salivary gland samples were genotyped to confirm sex. 100 ng of genomic DNA prepared as described above was PCR amplified using SapphireAMP master mix (Takara) following manufacturer’s instructions. Y-chromosome-specific *ARY* primers were 5’ TAGATACTTGGCGAGCAATGGA (forward) and 5’ ACCAAGAGGTGAAAAGGCTGTC (reverse) and PCR conditions were as follows: [94°C, 30s; 66°C touchdown, 30s (-0.5°C per cycle); 72°C, 5s]16x; [94°C, 30s; 58°C, 30s; 72°C, 5s]20x; 72°C, 5 min; held at 12°C. Samples were run on a 1.2% agarose gel, and those with a ~220 bp product were identified as males.

### Statistical analysis and graphical representation

Statistics were determined and graphs developed using GraphPad Prism (v. 10.1). Experiments examining only one variable (i.e. heat-shock versus no heat-shock absolute HR) used a student’s unpaired T-test with Welch’s correction or Wilcoxon paired T-test (i.e., HR vs. NHEJ proportions) for statistical analysis. To analyze the premeiotic germline data, whole fly TIDE data, larva TIDE data, and embryo TIDE data, a two-way ANOVA followed by Tukey’s multiple comparisons test was performed. Analysis of TIDE data of the larval and adult brains between sexes was performed using a three-way ANOVA followed by Tukey’s multiple comparisons test. Illustrations created with BioRender.com (license agreement number JK26O0ZS57).

## Results

### Impact of developmental stage and tissue type on DSB repair pathway choice

To examine the influence of developmental stage on DNA double-strand break repair pathway choice in multicellular tissues, we utilized the unique feature of the *Drosophila* embryo, which maintains both canonical (G1/S/G2/M) and non-canonical cell cycles. The first 13 embryonic divisions (0-3h post-egg deposition) consist of syncytial divisions with alternation between the S and M phases (S/M) and are completely controlled by maternal gene expression. At cellularization, three hours post-egg deposition, zygotic gene expression is initiated and the G2 phase is added (S/G2/M) to comprise the three post-blastoderm divisions. Six hours after fertilization, the G1 phase is added and the canonical cell cycle (G1/S/G2/M) is observed for the first time in many of the embryonic cells [28].

**Fig 1:**
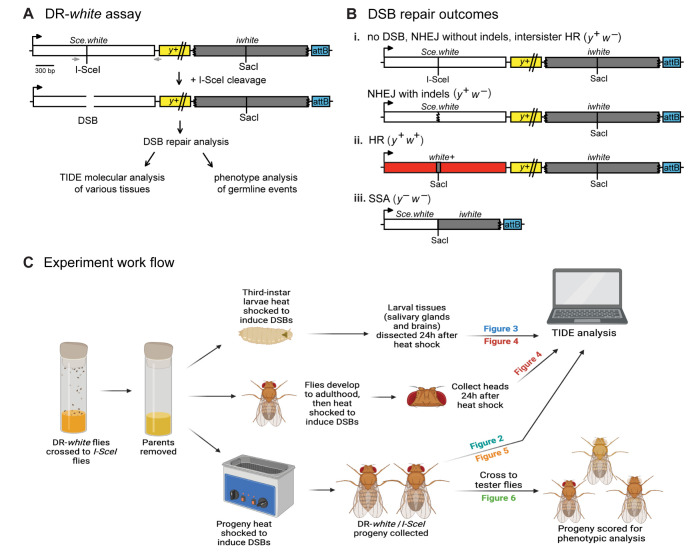
The DR-*white* DNA double-strand break (DSB) reporter assay. A. DR-*white* contains two nonfunctional direct repeats of the *white* gene. The first copy, *Sce*.*white*, is nonfunctional due to the insertion of the 18-bp I-SceI recognition sequence. The second copy, *iwhite*, is nonfunctional because of truncations at the 5’ and 3’ ends. When DR-*white* flies are crossed to flies expressing the *I-SceI* transgene, a double-strand break is created at the I-SceI site followed by repair. Repair events are analyzed molecularly through Tracking across Indels by DEcomposition (TIDE) via PCR amplification across the break site (gray arrows) and individual events of the premeiotic germline are analyzed by crossing F1 progeny to tester flies and scoring phenotypes of the progeny. B. Molecular and phenotypic outcomes of repair events may include: (i) no DSB, NHEJ without insertions or deletions (indels), intersister HR, or NHEJ with indels; (ii) homologous recombination; or (iii) single-strand annealing. C. Experimental work flow describing sample collection and processing for all heat-shock inducible *I-SceI* experiments. Illustration created with BioRender.com.

Females containing *Ubi*::*I-SceI* transgene (providing constitutive maternal and zygotic transcript expression of *I-SceI)* were crossed to males containing the established DSB repair reporter assay, DR-*white* (Fig 1) [12]. F1 embryos of this cross were aged and collected at various stages representing these three different cell cycles, allowing for the analysis of the impact of developmental stage on DSB repair. 0–3 hour-old embryos contain only maternal transcripts (including *I-SceI* transgene expression) and represent DSB repair during non-canonical cell cycles lacking G1 and G2 (S/M). 3–6 hour-old embryos include repair events from the S/M cycles and also include repair events that occurred in cells that have the G2 phase (lacking G1 phase; S/G2/M). Finally, 6–20 hour-old embryos include repair events of cells that have S/M cycles, S/G2/M, and also the canonical (G1/S/G2/M) cell cycle.

**Fig 2:**
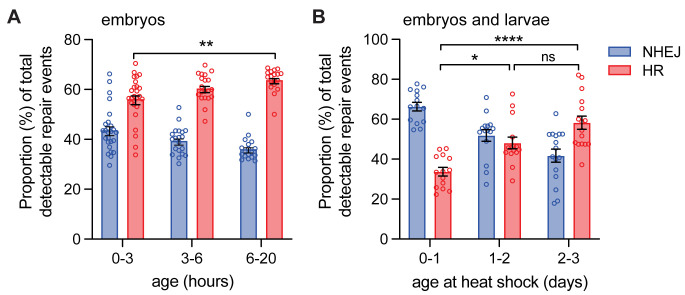
DSB repair pathway choice in embryos and larvae. A. *I-SceI* was expressed constitutively in DR-*white* embryos and collected at indicated ages and processed (*n* = 18–26). B. *I-SceI* was expressed via heat shock in DR-*white* embryos and larvae at indicated ages followed by processing once aged to adults (*n* = 14–16). Tracking across Indels by DEcomposition (TIDE) analysis was performed to determine the proportion of NHEJ with indels or HR of all detectable DSB repair events. Bars represent means; error bars are S.E.M. values. ns = not significant, *p < 0.05, **p < 0.01, ****p < 0.0001 by two-way ANOVA followed by Tukey’s multiple comparisons test.

Tracking across Indels by DEcomposition (TIDE) analysis of detectable repair events in embryos at these various developmental time points suggest that total detectable DSB repair increases with age (F_(2, 122)_ = 30.1; p <0.0001, by two-way ANOVA), for example from 65.2 ± 1.9% in 0-3h to 79.8 ± 2.1% in 6-20h (p <0.0001, Tukey’s multiple comparison test; S1A Fig). This increase in total repair is consistent with what was previously observed in germline analysis using constitutive expression of *I-SceI* [15]. When normalizing to detectable repair events, repair by HR increases in older embryos (F_(2, 122)_ = 13.3; p <0.0001, by two-way ANOVA; Fig 2A). In the first 0–3 hours after egg deposition, 43.8 ± 1.8% of the detectable repair events were through NHEJ and 56.2 ± 1.8% were through HR (Fig 2A). In the 3–6 hour time interval, 39.5 ± 1.2% of the detectable repair was through NHEJ and 60.5 ± 1.2% was via HR. Finally, for the 6–20 hour time interval, 36.1 ± 1.1% of the detectable repair events were NHEJ and 63.9 ± 1.1% of the repair was via HR (p <0.0001, Tukey’s multiple comparison test, Fig 2A). Thus, repair by HR is highest after 6 hours post-fertilization, with the initiation of the canonical cell cycle.

The increase in HR following the introduction of the canonical cell cycle at age 6h after egg deposition suggests that canonical-cycling cells preferentially repair DSBs via HR. To support this finding, DSBs were induced via heat-shock expression of *hsp70*.*I-SceI* in DR-*white* embryos (0–1 day old), first instar (1–2 day old), and second instar (2–3 day old) larvae, which contain canonical-cycling cells and some polyploid cells. The DSB repair outcomes were determined in adult males after the flies developed into adulthood. This whole fly analysis provided a sample of mostly mitotically-dividing somatic tissue, with a very small percentage of the tissue sample including the 16-cell mass of the male germlines [35]. While the absolute proportion of heat-shock induced detectable DSB repair events was lower than constitutive *I-SceI* expression (S1A and S1B Fig), it increased relative to non-heat-shocked controls (S1C and S1D Fig; 20.0 ± 3.0% for heat-shocked samples vs. 13.9 ± 1.0% for non-heat-shocked controls; p < 0.01, unpaired student’s T-test with Welch’s correction). When comparing heat shocked samples, the absolute proportion of detectable DSB repair events did not change within each age group (p > 0.05, Tukey’s multiple comparison test, S1B Fig). However, there was a significant shift to a higher proportion of HR events within the detectable repair events in 2–3 day old larva (58.3 ± 3.3%) compared to embryos (33.7 ± 2.1%; p < 0.0001, Tukey’s multiple comparison test, Fig 2B). The lower proportion of HR in embryos after heat-shock induced DSBs is consistent with lower HR in 0–3 hour-old embryos under constitutive *I-SceI* expression. There was no significant difference in the proportion of HR in 1–2 day old larvae or 2–3 day old larvae, suggesting that DSB repair pathway choice does not change with age of DSB induction in larvae. Overall, this experiment allowed for the analysis of developmental stage (embryo vs. larvae) as well as tissue type (embryonic vs. larval).

**Fig 3:**
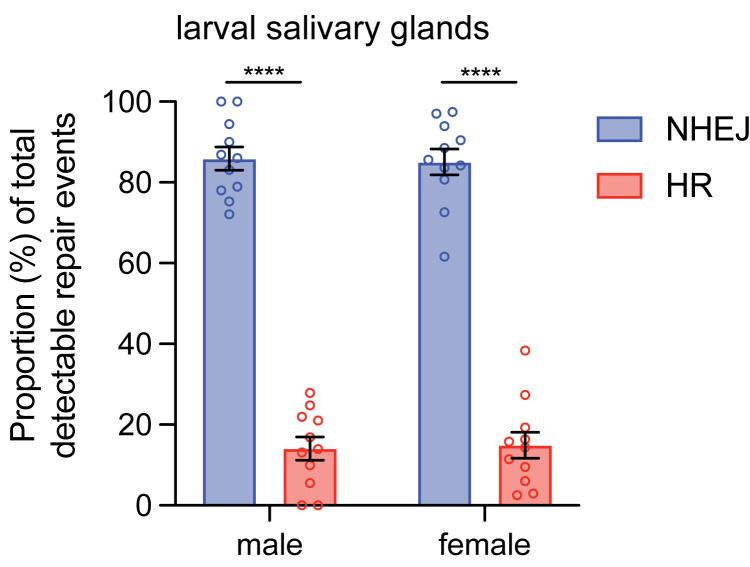
DSB repair pathway choice in larval salivary glands. *I-SceI* was expressed by heat shock in DR-*white* third instar larvae. 24 hours after heat shock, polyploid salivary glands were dissected and processed (*n* = 11 for both males and females). Tracking across Indels by DEcomposition (TIDE) analysis was performed to determine the proportion of NHEJ with indels or HR of all detectable DSB repair events. Bars represent means; error bars are S.E.M. values. ****p < 0.0001 by two-way ANOVA followed by Tukey’s multiple comparisons test. There is no significant difference when comparing the proportion of repair events between sexes (F_(1,40)_ = 0.705, p >0.99).

Our data suggest that repair by HR is maximized in tissues that are utilizing the canonical cell cycle. Although larvae contain mostly canonical-cycling cells, larval salivary glands cycle through the S and G phases without mitotic division (S/G). This provides a unique challenge in maintaining the genome, as there are hundreds of chromatids available for HR in these polyploid cells, yet re-replication due to endocycling often results in deletions in the salivary glands [36]. Similar to canonical-cycling cells, endocycling cells activate a marker for the DNA DSB response during replication damage, and can repair the damage through HR and NHEJ [37]. However, unlike canonical-cycling cells, damaged endocycling cells do not maintain caspase-mediated apoptosis [37]. This suggests that the DNA response in polyploid cells differs from that of embryos and may impact how DSB repair pathways are used in these cells. Thus, the larval salivary glands provide a unique environment to study the impact of tissue type and, by extension, cell cycle on repair. To analyze events in these larval polyploid cells, third instar male larvae (4–5 days old) containing DR-*white* and *hsp70*.*I-SceI* were heat shocked, salivary glands were dissected 24 hours later, and DSB repair events were molecularly analyzed with TIDE. Of the detectable repair events analyzed, surprisingly, a majority of DSBs were repaired through NHEJ (85.9 ± 2.9%) compared to HR (14.1 ± 2.9%; p <0.0001 by Tukey’s multiple comparisons test; Fig 3).

**Fig 4:**
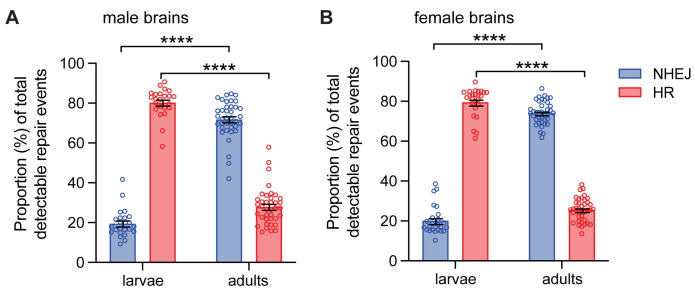
DSB repair pathway choice in male and female larval and adult tissues. *I-SceI* was expressed via heat shock in DR-*white* larvae and adults. A. Male larval brains (*n* = 23) and adult heads (*n* = 38) were molecularly analyzed by Tracking across Indels by DEcomposition (TIDE) to determine relative proportions of HR and NHEJ with indels. B. Female larval brains (*n* = 25) and adult brains (*n* = 38) were subject to molecular analysis by TIDE to determine relative proportions of HR and NHEJ with indels. Bars represent means; error bars are S.E.M. values. ****p < 0.0001 by three-way ANOVA followed by Tukey’s multiple comparisons test. There is no significant difference when comparing the proportion of repair events between sexes (F_(1,240)_ = 2.363, p >0.05).

The lower proportion of HR events in the larval salivary glands prompted an investigation to determine the relative proportion of HR and NHEJ repair in other tissues of larvae, in particular, canonical-cycling diploid cells such as neuroblasts of the larval brain [38]. Third-instar male larvae containing both DR-*white* and the *hsp70*.*I-SceI* transgene were heat shocked at 38°C for one hour to induce DSBs. 24 hours later, larval brains were dissected and brains were analyzed by TIDE. These tissues had a strikingly high proportion of HR repair (80.4 ±1.5%) and subsequent smaller proportion of repair events via NHEJ (19.6 ± 1.5%; p <0.0001 by Tukey’s multiple comparisons test; Fig 4A). This observation was consistent in other larval tissues, such as imaginal wing discs (63.6 ± 1.6% for HR and 36.4 ± 1.6% for NHEJ; p <0.01 by Wilcoxon paired T-test; S2 Fig).

The high proportion of HR in the canonical-cycling neuroblasts of the larval brain are similar to previous findings [34], suggesting that cycling cells preferentially repair DSBs via HR. To test this, we compared DSB repair events to similar tissues of the adult head, which contains mostly non-cycling cells [39]. 0–1 day old adult male flies containing both the DR-*white* reporter and *hsp70*.*I-SceI* were heat-shocked and samples were collected 24 hours later to molecularly analyze DSB repair in adult tissues. Male adults repaired a majority of DSBs through NHEJ with indels (71.9 ± 1.5%) compared to HR (28.1 ± 1.5%; Fig 4A). The absolute proportion of HR in adult brains was not different from that in non-heat-shocked controls (p > 0.05 by unpaired student’s T-test with Welch’s correction; S3 Fig). Comparison of the relative proportions of HR and NHEJ between male larvae and adults demonstrated a significant role of developmental stage and tissue type (larval v. adult) in repair pathway choice (F_(1,118)_ = 1,064; p < 0.0001 by two-way ANOVA; Fig 4A).

### Impact of sex on DSB pathway choice

Our data suggests that developmental stage and tissue type—and, consequently, whether the cell is non-cycling, canonically cycling, or non-canonically cycling—dramatically impact DSB repair pathway choice between HR and NHEJ in multicellular tissues. We next investigated whether sex impacted DSB repair outcomes. To investigate this, experiments on larval salivary glands, larval brains, and adult tissues were repeated in females. DSB repair in larval salivary glands also favored NHEJ in females (85.1 ± 3.2%) (Fig 3), although proportions of HR and NHEJ did not differ between females and males (F_(1,40)_ = 0.07; p > 0.99 by two-way ANOVA). In larval brains, repair in females was similar to that in males, demonstrating a higher proportion of HR (79.7 ± 1.5%) compared to adults (25.7 ± 0.9%) and a lower proportion of NHEJ (20.3 ± 1.5%) than adults (74.3 ± 0.9%) (Fig 4B). The absolute proportion of HR events in the female adult tissues was not different from the non-heat-shocked controls (p > 0.05 by unpaired student’s T-test with Welch’s correction; S3 Fig). Direct comparison of female larvae and adults also demonstrated significance of developmental stage and tissue type on repair pathway choice (F_(1,122)_ = 2140; p < 0.0001 by two-way ANOVA; Fig 4B). There were no sex-specific differences in repair pathway choice across larvae and adults (F_(1,240)_ = 2.363, p >0.05 by three-way ANOVA). Thus, there was no significant difference in DSB repair pathway choice between males and females in larval salivary glands, larval brains, or adult tissues.

**Fig 5:**
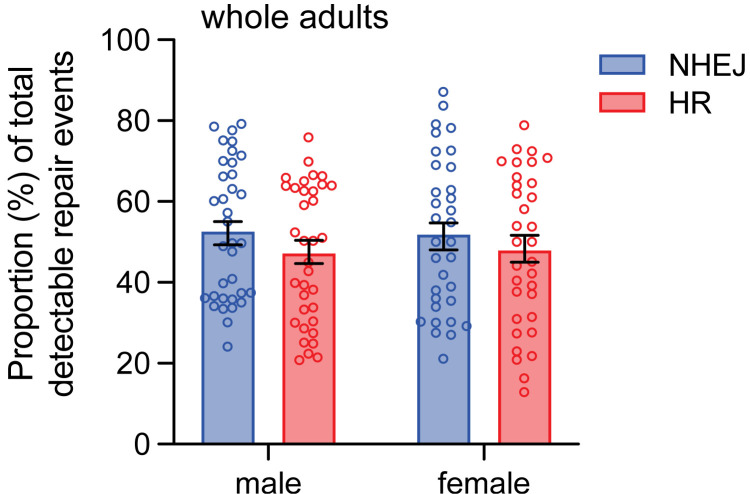
DSB repair pathway choice in male and female whole-fly tissue. *I-SceI* was expressed via heat shock in 0–3 day old DR-*white* larvae aged to adults. Whole flies (*n* = 35 males, 33 females) were molecularly analyzed using Tracking across Indels by DEcomposition (TIDE) to determine relative proportions of HR and NHEJ with indels. Bars represent means; error bars are S.E.M. values. There is no significant difference in the proportion of repair events within sexes (p > 0.05 by Tukey’s multiple comparisons test) or when comparing proportions of repair events between sexes (F_(1,132)_ = 1.57; p > 0.05 by two-way ANOVA with Tukey’s multiple comparisons test).

To further investigate the impact of sex on DSB repair pathway choice in other tissues, DSBs were induced in 0–3 day old embryos and larvae containing DR-*white* and *hsp70*.*I-SceI* and analyzed as adults with a comparison between males and females. In male and female whole flies, the proportions of HR and NHEJ were similar, with 47.3 ± 2.9% HR and 52.8 ± 2.9% NHEJ in males, and 48.1 ± 3.1% HR and 51.9 ± 3.1% NHEJ in females (Fig 5). There were no sex-specific differences in HR and NHEJ (F_(1,132)_ = 0.07; p > 0.05 by two-way ANOVA). There were also no differences in HR and NHEJ within the same sex (p > 0.05 by Tukey’s multiple comparisons test).

**Fig 6:**
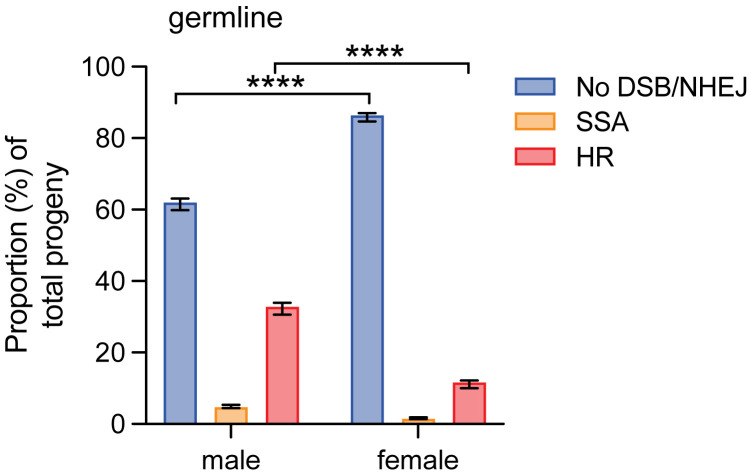
DSB repair in the male and female premeiotic germlines. F1 males and females containing DR-*white* and the *I-SceI* transgene were heat-shocked at 0–3 days old and aged to adults. These flies were crossed to *y w* tester flies of the opposite sex. Male (*n* = 66) and female (*n* = 59) premeiotic germlines were assessed by phenotypically analyzing their progeny (*n* = 5,894 total progeny for males and 2,759 total progeny for females) to determine the proportion of No DSB/NHEJ, SSA, and HR repair. Bars represent means; error bars are S.E.M. values. Statistics were determined with a two-way ANOVA (F_(2,384)_ = 179.3); ****p < 0.0001 by Tukey’s multiple comparisons test.

While there were no sex-specific differences in repair pathway choice in somatic tissue of larvae and adults, these differences may be more likely to be found in sex-specific tissues, such as the premeiotic germline. To determine whether sex impacts DSB repair pathway choice in the mitotically-dividing premeiotic germline, females containing the DR-*white* assay were crossed to males containing *hsp70*.*I-SceI*, and 0–3 day-old progeny were heat-shocked to induce *I-SceI* expression and create DSBs. F1 DR*-white/hsp70*.*I-SceI* adults of each sex were crossed to *y w* flies of the opposite sex and F2 progeny were phenotypically analyzed to determine the proportions of DSB repair by HR, No DSB/NHEJ, and SSA in male and female premeiotic germlines (Fig 1B). Comparing between sexes, there were significant differences in the premeiotic germline repair pathway choice (F_(2,384)_ = 179.3; p < 0.0001 by two-way ANOVA; Fig 6). Specifically, the amount of HR was lower in the female germline compared to the male germline (11.8 ± 1.1% compared to 33.0 ± 1.7%), with a concurrent higher proportion of no DSB/NHEJ in the female germline (86.6 ± 1.1% compared to 62.1 ± 1.6; p < 0.0001 by Tukey’s multiple comparisons test). SSA repair also decreased from the male premeiotic germline (4.9 ± 0.4%) to the female (1.6 ± 0.3%), although this difference was not significant (p > 0.05 by Tukey’s multiple comparisons test) (Fig 6).

## Discussion

To study the influence of developmental stage, tissue type, and sex on double-strand break (DSB) repair pathway choice, DSB repair in different tissues in male and female *Drosophila* was studied. The results demonstrate the importance of both developmental stage and tissue type, which offer different cell cycles for study, on DSB repair. In general, error-free repair by homologous recombination (HR) increased in cycling tissues that contain cells in the canonical G1/S/G2/M cell cycle (6–20 hour-old embryos, whole larvae, and larval brains). In contrast, HR was decreased in non-canonical-cycling cells of the embryo (0–3 hour old with S/M cycles), non-canonical G/S endocycling polyploid cells (larval salivary glands), and mostly terminally differentiated tissues (adult heads). None of these tissues demonstrated an influence of sex on repair outcomes when analyzed. However, a significant decrease in HR repair in the premeiotic female germline was observed compared to males.

The results from the developing embryo highlighted the importance of developmental stage on DSB repair. Particularly, the presence of different cell cycles throughout embryonic development underscored the impact of the canonical cell cycle in DSB repair. In the embryo, the total number of detectable repair events increased over time; this was expected if a DSB was repaired in a manner that maintained the I-SceI recognition site sequence (i.e., NHEJ without indels or intersister HR). These events could then be cleaved again due to constitutive *I-SceI* expression, which would allow for breaks to continue until a repair product that loses the I-SceI recognition sequence is achieved (i.e., intrachromosomal HR with gene conversion or NHEJ with indels). However, despite the total number of detectable repair events increasing, the proportion of HR out of all detectable repair events was not consistent in different embryonic stages, suggesting that DSBs induced during the later embryonic stages prefer repair by HR.

It is possible that the presence of the sister chromatid in the early (0-3h) embryos undergoing rapid S phase could lead to trans events utilizing the *Sce*.*white* sequence on the sister; however, these events would maintain the I-SceI recognition sequence and thus go undetected in this system. Additionally, our data suggest that a donor sequence *in cis* is highly preferred over the homologous sequence [9]. Thus, we conclude that the lower proportion of HR in detectable DSB repair events of blastoderm embryos is most likely due to the unique features at this developmental stage. The lower proportion of HR observed in the early S/M endocycling cells is consistent with the observation that homology-directed repair (HDR) of CRISPR/Cas9 DSBs is suppressed in rapidly dividing mammalian embryonic blastocysts [40]. This observation could be attributed to rapid cell cycles of ~9 minutes in the early syncytial cycles and only ~21 minutes before cellularization. After cellularization (~3 hours post egg deposition), the cell cycle is significantly prolonged (i.e., S phase increases from 10 minutes to 50 minutes with an additional G2 phase) [41]. Considering NHEJ is more kinetically efficient than HR in mammalian cells [42], this may lead to higher proportion of NHEJ during these rapid divisions in *Drosophila*. In addition, the early embryo contains a large number (50–100) of cells that develop into polyploid yolk cells of the pre-blastoderm embryo [28]. Considering other polyploid tissues suppress the DNA damage response [37] and suppress HR (this study), the polyploid population in the early embryo may also account for the larger proportion of NHEJ observed in 0–3 hour-old embryos.

We also observed an increase in HR when *I-SceI* was expressed by heat shock induction in larvae (1–2 and 2-3d) compared to embryos (0-1d). However, interpretation of this is guarded, as the repair events in this experiment may come from events later in development as cell populations were harvested as adults. However, between experimental groups, samples were aged in parallel, thus we interpret the changes in the repair distribution in embryos compared to larvae is most likely to the developmental stage in which the organisms were heat-shocked. This increase may be due to the absence of the embryonic syncytial dividing cells in the larva, supporting our hypothesis that cell cycles within tissues impact DSB repair pathway choice.

In support of the impact of polyploidy on DSB repair pathway choice, we observed a suppression of HR in larval salivary glands, which maintain S/G endocycles without mitosis. The suppression of a more error-free repair pathway may have significance in human tissues that maintain polyploidy state, such as liver hepatocytes, skin keratinocytes, and heart and skeletal muscle [43], where polyploidy plays an important role in human organogenesis and development [44]. It is possible that the suppression of HR in these tissues is to prevent hyperrecombination phenotypes that are hallmarks of genome instability. Thus, while NHEJ may inherently be more error prone, in tissues where aberrant recombination may occur with multiple homologous sequences available, it may be more stable to repair by NHEJ.

When analyzing the cycling larval brain tissue, it was expected that the proportion of HR would be higher than that of NHEJ, since cycling cells include both S and G2 phases when HR repair primarily occurs [45,46]. During these phases, a sister chromatid can serve as a template for repair. The NHEJ events observed may be due to DSBs that occur in the G1 phase of the cycling cells, or in the non-cycling glial cells (~5–10% of CNS) and ~2500 neurons found in larval brains [47,48]. However, when examining cycling imaginal discs, we observed nearly one third of detectable repair events are characterized as NHEJ with indels, suggesting that tissues containing cycling cells do repair by NHEJ, albeit to a lesser extent than HR. In contrast, most cells in the adult head (including the brain) are terminally differentiated and thus do not have an S or G2 phase with a sister chromatid available as a repair template. While HR can occur on a homologous chromosome template, it has been established that the sister chromatid is the preferred template [9,11,49]. As such, postmitotic terminally differentiated neuronal cells repair the majority of their breaks by NHEJ [50,51].

It has been demonstrated that a small population of cells in the adult brain of several model organisms, including *Drosophila*, are polyploid to help protect against the negative effects of DNA damage [52,53]. While this cell type accumulates in the brain with age, less than 5% of the cells are polyploid at the age of our adult brain samples [53]. Additionally, there is a small population of cells in the adult brain that are cycling, but the cycling clones only make up ~3% of the adult brain and are likely adult stem cells [38,39]. Thus, these tissues can be analyzed as primarily postmitotic cells that demonstrate a strong proportion of NHEJ to repair DSBs induced in adults.

Despite the preference for NHEJ in non-cycling cells, there was a small proportion of HR repair events in non-cycling tissues. However, the HR observed in non-dividing adult tissues may be the result of repair events occurring earlier in development from leaky expression of the *hsp70*.*I-SceI* transgene (i.e., expression without heat shock induction). Namely, larval cells that were mitotically dividing may have repaired DSBs that occurred due to this leaky expression of *I-SceI* and subsequent DSB repair before developing into adult cells. In this case, HR is then observed in non-cycling tissues. Leaky expression of this transgene has been observed previously [15] and is consistent with HR observed in non-heat-shocked controls (S3 Fig). While a small percentage of cells in the adult brain are either polyploid or cycling [39], which may account for some of the HR events observed, similar HR proportions with and without heat shock suggest that our observed HR is from leaky expression and not cell type.

Broadly, repair pathway choice has important implications for different tissues. Whole flies demonstrate a large proportion of repair by HR, which is consistent with CRISPR/Cas9 induced DSB repair by somatic gene conversion [54]. However, they repair DSBs almost equally by NHEJ, which is surprising given that NHEJ can be error prone if ends are processed to include indels. However, NHEJ has been shown to be more efficient than HR during all phases of the cell cycle, even in S phase when HR is the predominant repair pathway [42]. It is proposed that preferential use of NHEJ may stem from the repetitive nature of the genome, which is highly repetitive in both *Drosophila* and humans, and the fact that an incorrect HR template could cause gross genomic rearrangements, whereas the small indels associated with NHEJ are less harmful [42]. NHEJ is also faster in mammalian cells, taking an average of 30 minutes, while HR takes at least seven hours [42]. Thus, the organism may have a compelling and biologically-relevant reason to repair a large number of breaks via NHEJ, despite the risk for associated indels that change the genetic sequence at the break site.

The significant decrease in HR in the female premeiotic germline may have biological relevance. In the male premeiotic germline, a large percentage of detectable repair events are repaired by HR [12], suggesting that the error-free pathway is important in the *Drosophila* male germline. For gametocytes, error-free repair may be more important, as these tissues are responsible for heredity and often commit apoptosis instead of risking passing on deleterious mutations [54]. In the female germline, we suggest that cells containing DSBs destined for HR repair are lost through apoptosis. The difference between males and females could be due to differences in stress responses in the two germlines. In male *Drosophila* germline stem cells, the anti-apoptotic factor DIAP1 is responsible for preventing cell death in response to stress, and its upregulation protects adjacent spermatogonia [55]. It may be that the female germline lacks these protective factors and is thus more susceptible to stress-induced apoptosis.

In the meiotically-dividing female germline, i.e. oocytes, previous work has demonstrated a deficient DNA damage response. Namely, the G2 checkpoint that is normally established in cells following DNA damage fails to be efficiently activated, and oocytes continue into M phase [56]. Although the oocytes detect the DNA damage, they do not activate the ATM kinase required for cell cycle arrest [57], and damage levels must be severe for repair to occur [58]. However, during meiotic arrest in prophase I, p63 can induce oocyte arrest and initiate the apoptotic program in the presence of DNA damage [59]. Interestingly, in a mouse model, inhibition of apoptosis (achieved by using *Tp63*^-/-^ mice) in prophase-arrested oocytes increased the repair of DSBs by HR [60].

Considering these findings in the meiotically-dividing germline, we hypothesize that the decrease in HR in the mitotically-dividing premeiotic germline may also be due to apoptosis mediated by the human p63 paralog, *Drosophila* p53, which is similar to human *p53* [61]. Related, the apoptotic program in response to somatic DNA damage in *Drosophila* is driven by p53 [62] and the p53A isoform has been shown to be necessary and sufficient for inducing the apoptotic program in the mitotically-dividing germline [63]. Of note, given the extensive damage required to initiate a checkpoint, one break per cell (as occurs in the *I-SceI* experimental system used to study the premeiotic germline) may not be sufficient to initiate p53-induced apoptosis. Thus, an experiment in a different system, such as one under constitutive *I-SceI* expression that would allow for breaks to persist until a terminal event occurs, may more effectively address this hypothesis.

While work in other model systems and in *Drosophila* support the p53-mediated apoptosis hypothesis, an alternative interpretation to the decrease in HR in the female premeiotic germline is that there is a decrease in DSB formation or that the DSBs are repaired in an error-free NHEJ mechanism via a yet to be identified mechanism. Lastly, DSB repair may persist beyond the premeiotic germline (e.g., *I-SceI* transcripts that may persist after homologs segregate in meiosis I) which may impact repair outcomes. Studies limiting expression of *I-SceI* to the premeiotic germline may provide further insight to the effect of sex on repair in germline tissues [64]. Further studies are required to elucidate the role that *Drosophila* p53 (or other factors) may play in suppressing HR repair in females. Our findings warrant the investigation of the influence of sex in other tissues and organisms in order to develop a more nuanced understanding of the factors that influence repair pathway choice in multicellular organisms.

## Supporting information

S1 Fig Proportion of all DSB repair events in embryos and larvae. A. *I-SceI* was expressed constitutively and collected at indicated ages and immediately processed. B. *I-SceI* was expressed via heat shock at indicated ages followed by processing once aged to adults. C and D. Flies containing heat shock inducible *I-SceI* and DR-*white* were not heat-shocked as a control to examine leaky expression of the *I-SceI* transgene (i.e. expression of the transgene without heat-shock induction). Tracking across Indels by DEcomposition (TIDE) analysis was used to determine no repair (No DSB), total detectable repair (NHEJ with indels + HR), absolute repair by NHEJ with indels, and absolute repair by HR. Error bars represent SEM; ns = not significant, **p < 0.01, ****p < 0.0001 by two-way ANOVA followed by Tukey’s multiple comparisons test.
(EPS)

S2 Fig DSB repair pathway choice in larval imaginal discs. *I-SceI* was expressed via heat shock in male third-instar DR-*white* larvae. Imaginal wing discs (*n* = 8) were molecularly analyzed by Tracking across Indels by DEcomposition (TIDE) to determine relative proportions of HR and NHEJ with indels. Bars represent means; error bars are S.E.M. values. **p < 0.01 by Wilcoxon paired T-test.
(EPS)

S3 Fig Absolute HR repair in experimental samples and non-heat-shocked controls. Adult brains were either heat-shocked in experimental conditions to analyze repair in the adult brain or not heat-shocked as a control to examine leaky expression of the *I-SceI* transgene (i.e. expression of the transgene without heat-shock induction). The absolute percentage of HR was compared between the experimental and control adult brains using Tracking across Indels by DEcomposition (TIDE). Bars represent means; error bars are S.E.M values. There is no significant difference within each sex when comparing absolute % of HR repair events between with or without heat shock (p > 0.05 by unpaired student’s T-test with Welch’s correction).
(EPS)

S1 Data Raw data values from all experiments. (XLSX)

## References

[1] Yao Y, Dai W. Genomic Instability and Cancer. J Carcinog Mutagen. 2014;5: 1000165. doi: 10.4172/2157-2518.1000165 25541596 PMC4274643

[2] Ui A, Chiba N, Yasui A. Relationship among DNA double-strand break (DSB), DSB repair, and transcription prevents genome instability and cancer. Cancer Sci. 2020;111: 1443–1451. doi: 10.1111/cas.14404 32232911 PMC7226179

[3] Moynahan ME, Jasin M. Mitotic homologous recombination maintains genomic stability and suppresses tumorigenesis. Nat Rev Mol Cell Biol. 2010;11: 196–207. doi: 10.1038/nrm2851 20177395 PMC3261768

[4] Weterings E, van Gent DC. The mechanism of non-homologous end-joining: a synopsis of synapsis. DNA Repair (Amst). 2004;3: 1425–1435. doi: 10.1016/j.dnarep.2004.06.003 15380098

[5] Bhargava R, Onyango DO, Stark JM. Regulation of Single-Strand Annealing and its Role in Genome Maintenance. Trends Genet. 2016;32: 566–575. doi: 10.1016/j.tig.2016.06.007 27450436 PMC4992407

[6] Wright WD, Shah SS, Heyer W. Homologous recombination and the repair of DNA double-strand breaks. J Biol Chem. 2018;293: 10524–10535. doi: 10.1074/jbc.TM118.000372 29599286 PMC6036207

[7] LaRocque JR, Jasin M. Mechanisms of recombination between diverged sequences in wild-type and BLM-deficient mouse and human cells. Mol Cell Biol. 2010;30: 1887–1897. doi: 10.1128/MCB.01553-09 20154148 PMC2849462

[8] Szostak JW, Orr-Weaver TL, Rothstein RJ, Stahl FW. The double-strand-break repair model for recombination. Cell. 1983;33: 25–35. doi: 10.1016/0092-8674(83)90331-8 6380756

[9] Fernandez J, Bloomer H, Kellam N, LaRocque JR. Chromosome Preference During Homologous Recombination Repair of DNA Double-Strand Breaks in Drosophila melanogaster. G3 (Bethesda). 2019;9: 3773–3780. doi: 10.1534/g3.119.400607 31519746 PMC6829126

[10] Johnson RD, Jasin M. Double-strand-break-induced homologous recombination in mammalian cells. Biochem Soc Trans. 2001;29: 196–201. doi: 10.1042/0300-5127:0290196 11356153

[11] Kadyk LC, Hartwell LH. Sister chromatids are preferred over homologs as substrates for recombinational repair in Saccharomyces cerevisiae. Genetics. 1992;132: 387–402. doi: 10.1093/genetics/132.2.387 1427035 PMC1205144

[12] Do AT, Brooks JT, Le Neveu MK, LaRocque JR. Double-strand break repair assays determine pathway choice and structure of gene conversion events in Drosophila melanogaster. G3 (Bethesda). 2014;4: 425–432. doi: 10.1534/g3.113.010074 24368780 PMC3962482

[13] Heyer WD, Ehmsen KT, Liu J. Regulation of homologous recombination in eukaryotes. Annu Rev Genet. 2010;44: 113–139. doi: 10.1146/annurev-genet-051710-150955 20690856 PMC4114321

[14] San Filippo J, Sung P, Klein H. Mechanism of Eukaryotic Homologous Recombination. Annual Review of Biochemistry. 2008;77: 229–257. doi: 10.1146/annurev.biochem.77.061306.125255 18275380

[15] Delabaere L, Ertl HA, Massey DJ, Hofley CM, Sohail F, Bienenstock EJ, et al. Aging impairs double-strand break repair by homologous recombination in Drosophila germ cells. Aging Cell. 2017;16: 320–328. doi: 10.1111/acel.12556 28000382 PMC5334535

[16] Shrivastav M, De Haro LP, Nickoloff JA. Regulation of DNA double-strand break repair pathway choice. Cell Res. 2008;18: 134–147. doi: 10.1038/cr.2007.111 18157161

[17] Aleksandrov R, Hristova R, Stoynov S, Gospodinov A. The Chromatin Response to Double-Strand DNA Breaks and Their Repair. Cells. 2020;9: 1853. doi: 10.3390/cells9081853 32784607 PMC7464352

[18] Sekelsky J. DNA Repair in Drosophila: Mutagens, Models, and Missing Genes. Genetics. 2017;205: 471–490. doi: 10.1534/genetics.116.186759 28154196 PMC5289830

[19] Ceccaldi R, Rondinelli B, D’Andrea AD. Repair Pathway Choices and Consequences at the Double-Strand Break. Trends Cell Biol. 2016;26: 52–64. doi: 10.1016/j.tcb.2015.07.009 26437586 PMC4862604

[20] Mathiasen DP, Lisby M. Cell cycle regulation of homologous recombination in Saccharomyces cerevisiae. FEMS Microbiol Rev. 2014;38: 172–184. doi: 10.1111/1574-6976.12066 24483249

[21] Mao Z, Bozzella M, Seluanov A, Gorbunova V. DNA repair by nonhomologous end joining and homologous recombination during cell cycle in human cells. Cell Cycle. 2008;7: 2902–2906. doi: 10.4161/cc.7.18.6679 18769152 PMC2754209

[22] Plevkova J, Brozmanova M, Harsanyiova J, Sterusky M, Honetschlager J, Buday T. Various aspects of sex and gender bias in biomedical research. Physiological research. 2020;69: S367–S378. doi: 10.33549/physiolres.934593 33464920 PMC8603716

[23] Woitowich NC, Beery A, Woodruff T. A 10-year follow-up study of sex inclusion in the biological sciences. eLife. 2020;9: e56344. doi: 10.7554/eLife.56344 32513386 PMC7282816

[24] Beery AK, Zucker I. Sex bias in neuroscience and biomedical research. Neurosci Biobehav Rev. 2011;35: 565–572. doi: 10.1016/j.neubiorev.2010.07.002 20620164 PMC3008499

[25] Ranz JM, Castillo-Davis CI, Meiklejohn CD, Hartl DL. Sex-dependent gene expression and evolution of the Drosophila transcriptome. Science. 2003;300: 1742–1745. doi: 10.1126/science.1085881 12805547

[26] Hughes SE, Miller DE, Miller AL, Hawley RS. Female Meiosis: Synapsis, Recombination, and Segregation inDrosophila melanogaster. Genetics. 2018;208: 875. doi: 10.1534/genetics.117.300081 29487146 PMC5844340

[27] Mank JE. Sex-specific morphs: the genetics and evolution of intra-sexual variation. Nat Rev Genet. 2023;24: 44–52. doi: 10.1038/s41576-022-00524-2 35971002

[28] Orr-Weaver TL. Developmental modification of the Drosophila cell cycle. Trends Genet. 1994;10: 321–327. doi: 10.1016/0168-9525(94)90035-3 [pii]. 7974746

[29] Rong YS, Golic KG. Gene targeting by homologous recombination in Drosophila. Science. 2000;288: 2013–2018. doi: 10.1126/science.288.5473.2013 10856208

[30] Preston CR, Flores CC, Engels WR. Differential usage of alternative pathways of double-strand break repair in Drosophila. Genetics. 2006;172: 1055–68. doi: 10.1534/genetics.105.050138 16299390 PMC1456205

[31] Williams College, producer. Lebestky T and Louie R, directors. Larval Brain Dissection in Drosophila melanogaster; 2012: YouTube.

[32] Ertl HA, Russo DP, Srivastava N, Brooks JT, Dao TN, LaRocque JR. The Role of Blm Helicase in Homologous Recombination, Gene Conversion Tract Length, and Recombination Between Diverged Sequences in Drosophila melanogaster. Genetics. 2017;207: 923–933. doi: 10.1534/genetics.117.300285 28912341 PMC5676224

[33] Brinkman EK, Chen T, Amendola M, van Steensel B. Easy quantitative assessment of genome editing by sequence trace decomposition. Nucleic Acids Res. 2014;42: e168. doi: 10.1093/nar/gku936 25300484 PMC4267669

[34] Janssen A, Breuer GA, Brinkman EK, van der Meulen AI, Borden SV, van Steensel B, et al. A single double-strand break system reveals repair dynamics and mechanisms in heterochromatin and euchromatin. Genes Dev. 2016;30: 1645–1657. doi: 10.1101/gad.283028.116 27474442 PMC4973294

[35] Matunis EL, Stine RR, de Cuevas M. Recent advances in Drosophila male germline stem cell biology. Spermatogenesis. 2012;2: 137–144. doi: 10.4161/spmg.21763 23087833 PMC3469437

[36] Yarosh W, Spradling AC. Incomplete replication generates somatic DNA alterations within Drosophila polytene salivary gland cells. Genes Dev. 2014;28: 1840–1855. doi: 10.1101/gad.245811.114 25128500 PMC4197960

[37] Bretscher HS, Fox DT. Proliferation of Double-Strand Break-Resistant Polyploid Cells Requires Drosophila FANCD2. Dev Cell. 2016;37: 444–457. doi: 10.1016/j.devcel.2016.05.004 27270041 PMC4901310

[38] Li G, Hidalgo A. Adult Neurogenesis in the Drosophila Brain: The Evidence and the Void. Int J Mol Sci. 2020;21: 6653. doi: 10.3390/ijms21186653 32932867 PMC7554932

[39] von Trotha JW, Egger B, Brand AH. Cell proliferation in the Drosophila adult brain revealed by clonal analysis and bromodeoxyuridine labelling. Neural Dev. 2009;4: 9. doi: 10.1186/1749-8104-4-9 19254370 PMC2662830

[40] Wilde JJ, Aida T, Del Rosario RCH, Kaiser T, Qi P, Wienisch M, et al. Efficient embryonic homozygous gene conversion via RAD51-enhanced interhomolog repair. Cell. 2021;184: 3267–3280.e18. doi: 10.1016/j.cell.2021.04.035 34043941 PMC8240950

[41] Yuan K, Seller CA, Shermoen AW, O’Farrell PH. Timing the Drosophila Mid-Blastula Transition: a cell cycle-centered view. Trends Genet. 2016;32: 496–507. doi: 10.1016/j.tig.2016.05.006 27339317 PMC4958567

[42] Mao Z, Bozzella M, Seluanov A, Gorbunova V. Comparison of nonhomologous end joining and homologous recombination in human cells. DNA Repair (Amst). 2008;7: 1765–1771. doi: 10.1016/j.dnarep.2008.06.018 18675941 PMC2695993

[43] Peterson NG, Fox DT. Communal living: the role of polyploidy and syncytia in tissue biology. Chromosome Res. 2021;29: 245–260. doi: 10.1007/s10577-021-09664-3 34075512 PMC8169410

[44] Orr-Weaver TL. When bigger is better: the role of polyploidy in organogenesis. Trends in Genetics. 2015;31: 307–315. doi: 10.1016/j.tig.2015.03.011 25921783 PMC4537166

[45] Huertas P, Jackson SP. Human CtIP mediates cell cycle control of DNA end resection and double strand break repair. J Biol Chem. 2009;284: 9558–9565. doi: 10.1074/jbc.M808906200 19202191 PMC2666608

[46] Yun MH, Hiom K. CtIP-BRCA1 modulates the choice of DNA double-strand-break repair pathway throughout the cell cycle. Nature. 2009;459: 460–463. doi: 10.1038/nature07955 19357644 PMC2857324

[47] Eschbach C, Zlatic M. Useful road maps: studying Drosophila larva’s central nervous system with the help of connectomics. Curr Opin Neurobiol. 2020;65: 129–137. doi: 10.1016/j.conb.2020.09.008 33242722 PMC7773133

[48] Freeman MR. Drosophila Central Nervous System Glia. Cold Spring Harb Perspect Biol. 2015;7: a020552. doi: 10.1101/cshperspect.a020552 25722465 PMC4632667

[49] Johnson RD, Jasin M. Sister chromatid gene conversion is a prominent double-strand break repair pathway in mammalian cells. Embo J. 2000;19: 3398–407. doi: 10.1093/emboj/19.13.3398 10880452 PMC313931

[50] Dobbin MM, Madabhushi R, Pan L, Chen Y, Kim D, Gao J, et al. SIRT1 collaborates with ATM and HDAC1 to maintain genomic stability in neurons. Nat Neurosci. 2013;16: 1008–1015. doi: 10.1038/nn.3460 23852118 PMC4758134

[51] Schneider L, Fumagalli M, d’Adda di Fagagna F. Terminally differentiated astrocytes lack DNA damage response signaling and are radioresistant but retain DNA repair proficiency. Cell Death Differ. 2012;19: 582–591. doi: 10.1038/cdd.2011.129 21979466 PMC3307974

[52] Nandakumar S, Rozich E, Buttitta L. Cell Cycle Re-entry in the Nervous System: From Polyploidy to Neurodegeneration. Front Cell Dev Biol. 2021;9: 698661. doi: 10.3389/fcell.2021.698661 34249947 PMC8264763

[53] Nandakumar S, Grushko O, Buttitta LA. Polyploidy in the adult Drosophila brain. eLife. 2020;9. doi: 10.7554/eLife.54385 32840209 PMC7447450

[54] Bloom JC, Loehr AR, Schimenti JC, Weiss RS. Germline Genome Protection: Implications for Gamete Quality and Germ Cell Tumorigenesis. Andrology. 2019;7: 516–526. doi: 10.1111/andr.12651 31119900 PMC6635098

[55] Hasan S, Hétié P, Matunis EL. Niche signaling promotes stem cell survival in the Drosophila testis via the JAK–STAT target DIAP1. Developmental Biology. 2015;404: 27–39. doi: 10.1016/j.ydbio.2015.04.017 25941003 PMC4469572

[56] Subramanian GN, Greaney J, Wei Z, Becherel O, Lavin M, Homer HA. Oocytes mount a noncanonical DNA damage response involving APC-Cdh1–mediated proteolysis. The Journal of cell biology. 2020;219: 1. doi: 10.1083/jcb.201907213 32328643 PMC7147104

[57] Marangos P, Carroll J. Oocytes Progress beyond Prophase in the Presence of DNA Damage. Current Biology. 2012;22: 989–994. doi: 10.1016/j.cub.2012.03.063 22578416

[58] Leem J, Kim J, Oh JS. WIP1 phosphatase suppresses the DNA damage response during G2/prophase arrest in mouse oocytes. Biol Reprod. 2018;99: 798–805. doi: 10.1093/biolre/ioy108 29733326

[59] Kerr JB, Hutt KJ, Michalak EM, Cook M, Vandenberg CJ, Liew SH, et al. DNA damage-induced primordial follicle oocyte apoptosis and loss of fertility require TAp63-mediated induction of Puma and Noxa. Mol Cell. 2012;48: 343–352. doi: 10.1016/j.molcel.2012.08.017 23000175 PMC3496022

[60] Stringer JM, Winship A, Zerafa N, Wakefield M, Hutt K. Oocytes can efficiently repair DNA double-strand breaks to restore genetic integrity and protect offspring health. Proc Natl Acad Sci U S A. 2020;117: 11513–11522. doi: 10.1073/pnas.2001124117 32381741 PMC7260990

[61] Ollmann M, Young LM, Di Como CJ, Karim F, Belvin M, Robertson S, et al. Drosophila p53 is a structural and functional homolog of the tumor suppressor p53. Cell. 2000;101: 91–101. doi: 10.1016/S0092-8674(00)80626-1 10778859

[62] LaRocque JR, Dougherty DL, Hussain SK, Sekelsky J. Reducing DNA Polymerase α in the Absence of Drosophila ATR Leads to P53-Dependent Apoptosis and Developmental Defects. Genetics. 2007;176: 1441–1451. doi: 10.1534/genetics.107.073635 17483406 PMC1931523

[63] Chakravarti A, Thirimanne HN, Brown S, Calvi BR. Drosophila p53 isoforms have overlapping and distinct functions in germline genome integrity and oocyte quality control. eLife. 2022;11: e61389. doi: 10.7554/eLife.61389 35023826 PMC8758136

[64] Miller JM, Prange S, Ji H, Rau AR, Khodaverdian VY, Li X, et al. Alternative end-joining results in smaller deletions in heterochromatin relative to euchromatin. eLife. 2023;12. doi: 10.1101/2023.03.03.531058 37645729 PMC10461932

